# Care for patients living with chronic conditions using the ICAN Discussion Aid: A mixed methods cluster-randomized trial

**DOI:** 10.1371/journal.pone.0314605

**Published:** 2024-12-04

**Authors:** Kasey R. Boehmer, Anjali Thota, Paige Organick-Lee, Megan Branda, Alex Lee, Rachel Giblon, Emma Behnken, Hazel Tapp, Carl May, Victor Montori

**Affiliations:** 1 Knowledge and Evaluation Research (KER) Unit, Mayo Clinic, Rochester, Minnesota, United States of America; 2 Division of Health Care Delivery Research, Mayo Clinic, Rochester, Minnesota, United States of America; 3 School of Medicine, St. George’s University, University Centre Grenada, West Indies, Grenada; 4 George Washington University Milken Institute School of Public Health Graduate School, Washington, D.C., United States of America; 5 Division of Clinical Trials and Biostatistics, Mayo Clinic, Rochester, Minnesota, United States of America; 6 Dalla Lana School of Public Health, University of Toronto, Toronto, Ontario, Canada; 7 Department of Family Medicine, Atrium Health, Charlotte, North Carolina, United States of America; 8 Faculty of Public Health and Policy, London School of Hygiene and Tropical Medicine, London, United Kingdom; Guizhou Medical University, CHINA

## Abstract

**Objectives:**

To assess the effectiveness of the ICAN Discussion Aid in improving patients’ experience of receiving care for their chronic conditions and health professionals’ experience of providing their care.

**Methods:**

We conducted a pragmatic, mixed-methods, cluster-randomized trial of the ICAN Discussion Aid at 8 clinics in 4 independent health systems in the US from January 2017 and to August 2018. Sites were randomized 1:1 in pairs. Participants were primary care health professionals and their adult patients with ≥1 chronic condition. Quantitative outcomes were health professional assessment of chronic illness care and relational coordination and patient-reported self-efficacy to manage chronic disease, self-efficacy to communicate with clinician, treatment burden, assessment of chronic illness care, general health, and disruption from illness and treatment. Uptake of ICAN was assessed with patient qualitative interviews, clinician focus groups/interviews, visit video recordings, and chart review.

**Results:**

98 clinicians and 1733 patients participated. We found no significant differences between ICAN and usual care sites in mixed effect models on main outcome measures. In adjusted difference-in-differences analyses, we found patient self-efficacy to manage chronic disease (mean difference 0.61 (SE 0.27), p = 0.023), patient self-efficacy to communicate with their clinician (mean difference 0.31 (SE 0.14), p = 0.032), and health professional assessment of chronic illness care (1.42 (SE 0.52), p = 0.007) were significantly better at ICAN sites. Chart review indicated the aid was implemented in 19% of eligible encounters. Qualitative analyses highlighted limited implementation of ICAN as intended overall due to varying clinic challenges but showed that ICAN use as intended was a valued addition to the visit.

**Conclusions:**

When patients and clinicians use ICAN as intended, which seldom occurred, important conversations emerge. This qualitative finding did not parlay into statistically significant effects on most outcomes of interest.

**Trial registration:**

The trial was registered at clinicaltrials.gov (# NCT03017196).

## Background

Chronic conditions now affect 6 in 10 Americans, with multiple chronic conditions affecting 4 in 10 [1]. 90% of US healthcare spending yearly is accounted for by those living with chronic conditions [2]. These figures capture a national problem, and one that is deeply impactful on people’s everyday lives. From the patient and family’s perspective, the management of a single condition can occupy approximately 2 hours of each day [3]. This “work” that patients and families do to manage chronic illness includes scheduling and attending healthcare appointments, self-monitoring, getting prescribed amounts of exercise, and managing condition-specific dietary needs [4].

Many tasks regarding managing of chronic illnesses go unaccomplished; it is estimated that 30–50% of patients are unable to adhere to treatment and self-management routines [5, 6]. Furthermore, 40% of patients across condition types report that their current treatment regimens are unsustainable in the long-term [7]. This happens because the available capacity–time, energy, resources–is insufficient for patients to enact that work [4]. As workload exceeds capacity, treatment burden accumulates and the ability to access and use healthcare, health outcomes, quality of life, and ability to enact self-care deteriorates [4]. Unsustainable treatment burden has been shown to be associated with non-adherence to treatment routines [8].

**Fig 1:**
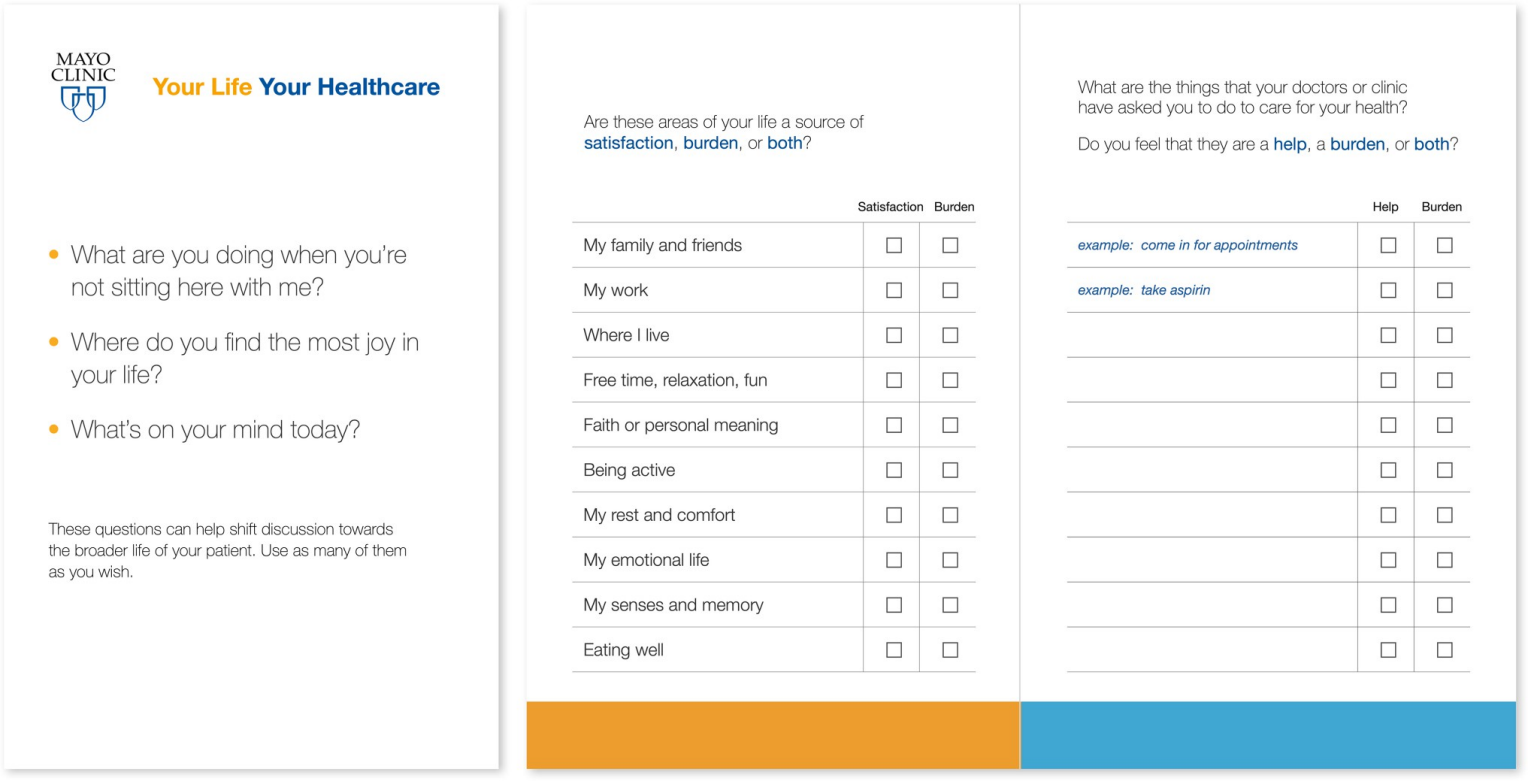
The ICAN Discussion Aid, original version.

Therefore, to improve patient outcomes and quality of life, we need ways to assess and address current levels of patient work and capacity before patients and families become overwhelmed. The ICAN Discussion Aid is a point-of-visit conversation aid developed to meet this need (Fig 1) [9]. ICAN was designed using a robust user-centered design process, which began with observations of chronic care clinical visits to understand the extent to which patient healthcare work and capacity were discussed [9]. Following observations, prototypes were developed and tested in clinical visits to determine their utility in better supporting these conversations [9]. Prototype iteration continued until aspects of patient workload and capacity were discussed within observed visits; a total of 74 observations occurred [9]. Following development, the finalized ICAN discussion aid was pilot tested in primary care clinics in a single healthcare system [10]. In the pilot, an in-depth videographic analysis of 45 ICAN visits compared to 39 usual care visits demonstrated that ICAN prompted different types of conversations, with issues of managing competing priorities, diet, being active, and medication taking coming up more often compared to usual primary care visits [10]. Furthermore, in post-visit surveys indicated that ICAN was deemed feasible to implement by practicing clinicians and did not increase time in the clinical visit, an often cited concern of introducing conversation aids into practice [10].

Building on this early work, we sought to test ICAN at diverse clinical sites in the US to assess its impact on patient perceptions of their clinical encounters, patient-reported treatment burden, and adherence to prescribed medication, as well as clinic provision of chronic care and staff perceptions.

## Methods

### Trial design

We conducted a pragmatic, mixed-methods, cluster-randomized trial, reported in accordance with the CONSORT reporting guidelines [11]. The Mayo Clinic Institutional Review Board (IRB) was the IRB of record for this multicenter trial for three participating health systems (# 16–007340), and Atrium Health IRB (# Pro00020992) approved procedures for their sites. The trial was registered at clinicaltrials.gov (# NCT03017196).

### Study sites

We enrolled two primary care clinics from each of four independent health care systems in the United States. Health systems were diverse across characteristics of geography (e.g. rural/urban), race/ethnicity distribution, median income of the community, and payment models (e.g. fee-for-service, capitation). Two systems were considered academic health systems and two were considered community health systems. Health systems, after agreeing to participate in the study, were given guidance to select two of their clinics that were similar in size and in makeup across these characteristics. Beyond the two sites being comparable across these characteristics, no additional guidance was given. The paired sites were randomized to ICAN or usual care.

### Randomization

Randomization occurred at the clinic level. After confirming eligibility and enrollment, the study’s statistician (M.B.) used a simple randomization to allocate a member of the pair of clinics to usual care and the other to implement the ICAN Discussion Aid (intervention). Unfortunately, two intervention sites dropped from the study either immediately prior to or right after training due to insufficient staff buy-in. Health system leaders identified replacement sites, but re-randomization was not possible because site visits and data collection had already commenced at control sites.

### Participants

All participants provided written consent for participation. At baseline visits, participating clinics provided a list of all clinicians at working at sites to research staff to be approached for consent. Clinicians, including physicians (MD/DO), advanced practice providers (PA/NP), nurses, clinical assistants, and health coaches working in participating practices were approached for participation.

Once clinicians had consented, research staff were provided with a daily list of patients being seen by consenting clinicians. Research staff worked with rooming staff to determine which patients met study eligibility criteria. Patients were eligible to participate if being seen by a consented clinician, were 18 years or older, and living with one or more chronic conditions. Patients were excluded if they were <18 years old, did not have a chronic condition, had barriers to consent such as dementia or cognitive impairment, or if they were unable to consent in English.

### Intervention

After enrollment and randomization, all clinics were visited by research staff to work with clinic leadership to sensibly embed the intervention in the clinic’s workflow. Intervention clinicians and office staff could choose to take part in a 1-3-hour training workshop as a group or individually. Training comprised of a review of ICAN’s purpose, a demonstration and coached use (with another clinician or with a patient volunteer), troubleshooting of workflow issues, and general discussion.

After the workshop, clinicians were encouraged to use ICAN routinely in their chronic care visits for at least a six-month period, which included three steps. First, desk or nursing staff distributed the paper based ICAN tool to patients during the rooming process. The patient was instructed to fill the tool for conversation with their clinician. The tool included two prompts and a checklist for responses: a) “are these areas of your life a satisfaction, burden, or both?” (e.g. my family and friends) and b) “what are the things that your doctors or clinic have asked you to do for your health? Do you feel that they are a help, a burden or both?” (e.g. come in for appointments). Second, upon entering the room for the clinical visit, the clinician was told to ask one of three questions a) “what are you doing when you’re not sitting here with me?” b) “Where do you find the most joy in your life?” or c) “what’s on your mind today?” Small reminder cards were taped near the clinician computers that showed these questions. Third, the clinician and patient looked together at the patient-filled card asking “what stands out to you from what you filled?”

## Quantitative data

### Data collection

Research staff visited sites for baseline and follow up visits each lasting two to three weeks. Baseline visits took place January–August 2017. Follow up visits took place at the practices’ convenience at least 6 months after baseline visits and were between December 2017 and August 2018. The first participant was enrolled on January 9, 2017, and the final participant was enrolled on August 29, 2018.

At visits to three of the four healthcare systems, Mayo Clinic researchers enrolled clinician and patient participants, and at the fourth system, Mayo Clinic staff trained local personnel to enrol participants. During site visits, all patient data was collected including survey responses, consent to review medical and pharmacy records, qualitative interviews, and visit video or audio-recordings collected. Given that study staff had to travel to the sites for a brief period to collect data, it was not possible to synchronize patient appointments to those periods. Thus, baseline and follow-up data come from different patient cohorts.

### Outcomes

Quantitative data was collected with in-person surveys from consented health professionals and in-person and postal surveys from consenting patients. The Patient Assessment of Chronic Illness Care was considered the primary outcome measure of the study; all other outcomes were secondary outcomes. Full scoring information is provided in S1 Appendix.

#### Health professional surveys

Health professional surveys were collected at the beginning of baseline visits and the conclusion of follow up visits. Surveys were designed to ascertain if clinic culture changed in response to implementation of ICAN and included:

- *Demographic Characteristics*: *clinician role*, *years in practice*, *age*, *gender*, *and % of panel with chronic conditions*
- *Assessment of Chronic Illness Care (ACIC)*: *a validated survey measure used to determine the extent of implementation of chronic care practices in alignment with the Chronic Care Model*, *including community linkages*, *self-management support*, *decision support*, *delivery system design*, *information systems*, *and organization of care* [12]
- *Relational Coordination* [13, 14]: *a validated and reliable measure* [15, 16], *to assesses seven domains of coordinated teams*: *frequent*, *timely*, *accurate*, *and problem-solving communication*, *shared goals*, *shared knowledge*, *and mutual respect*

#### Patient in-person surveys

Patient in-person surveys were collected after a consented patient completed their clinical visit with the consented clinician. Surveys included measures that we anticipated would be responsive to change if ICAN was used in a single visit with high fidelity to training. The following measures were included:

- *Demographic Characteristics*: *patient age*, *gender*, *ethnicity*, *race*, *marital status*, *employment status*, *annual household income*, *number of chronic conditions*.
- *Self-efficacy to Communicate with Clinician* *measured how confident patients are in communicating about their illness with their clinician* [17]
- *Self-efficacy to Manage Chronic Disease* *covered patient confidence in managing aspects of their illness* [17]
- *Treatment Burden* *assessed how burdened patients felt by their treatment regimens* [18]
- *Patient-Clinician Partnership* *measured using the Consultation Care Measure covering aspects of communication such as perceived clinician interest in the impact of the illness on their life* [19]

#### Patient postal surveys

Patient postal surveys were mailed patients of consented clinician that met the eligibility criteria if they had a visit with the clinician within the previous 6 months. Surveys included measures that we expected would be responsive to change if the clinics implemented ICAN routinely across intervention sites during the implementation period. The following scales were used in addition to the demographics above:

- *Patient Assessment of Chronic Illness Care* *assessed the patient-centeredness of care and alignment with the Chronic Care Model* [20]
- *General Health* *assessed with a single item from the SF-36* [21, 22]
- *Disruption from Illness and Treatment*, *assessed with the Illness Intrusiveness Scale* [23, 24], *covered how disrupted patients’ lives were from their illness and treatment*

#### Adherence

Patient pharmacy profiles were collected for patients enrolled for in-person participation at baseline and follow-up. Profiles consisted of all medication fills for six months prior to and following the consent date.

### Sample size

Our target enrollment was 1) 75 patients in-person per site for a total of 300 patients each in the intervention and control groups at baseline and follow-up; 2) 600 patients with postal surveys each in the intervention and control groups for baseline and follow-up. This equaled a total sample size of 1,800, which was determined in advance to be adequate for 80% power to detect clinically meaningful differences (0.5 standard deviations) in each survey outcome with a two-tailed test and an alpha of 0.05.

### Blinding

Blinding was not possible, as during enrollment sites knew they would be implementing a conversation aid or performing usual care. Further, the ICAN discussion aid was in use during outcome evaluator’s site visits, also preventing blinding. However, patients were not made aware of the intention of the study or the specific aspects of the intervention (e.g., they were asked to fill ICAN as part of the usual clinic workflow and were not aware of clinician-specific prompts about the discussion aid).

### Statistical methods

In accordance with intention-to-treat principals, all patients were analyzed in the arm in which they had been allocated. Demographics were summarized using frequencies and percentages for categorical variables and means with standard deviations for continuous variables. Comparisons for demographics were conducted using Kruskal-Wallis for continuous variables and the Chi-Square test for categorical unless the cell count is less than 5 the Fisher’s exact test. The guidelines for scoring and assessment for the Relational Coordination scale were applied and findings indicated that an average overall score for each responder was appropriate (Relational Coordination Analytics, Copyright 2016). All outcomes of interest are continuous and were assessed for normality. Mean differences between arms for continuous patient survey outcomes (in person and by postal) were modeled using hierarchical generalized linear models (HGLM) with random main effects specified at the site level adjusting for fixed effects of arm, timepoint, and the interaction between time point and arm, age, sex, race, ethnicity, and number of comorbid conditions. The analysis for clinician survey outcomes were modeled with a HGLM with random effects at the site level and adjusting for fixed effects of age, gender, type (Physician, Physician Assistant/RN, Other), years in practice (0–5, >5–10, >10–15, 15+) and percent of practice with chronic conditions (0–50%, >50–100%), including the Relational Coordination scale. The HGLMS for both patient and clinician outcomes were conducted as difference-in-difference (D-I-D) analysis to evaluate average intervention effects between arms over time. Medication adherence was analyzed by comparing the percent days covered (PDC) between arms 180 days prior and 180 days post patient encounter, an unadjusted analysis was conducted showing descriptive statistics. The PDC is calculated by taking the number of days that a person has access to a medication during the 180 days of interest, which is based on the fill dates and days’ supply for each dispensing medication of interest. The total days available is then divided by 180 to give us the proportion of days with the medication. Analysis was conducted on complete data, for outcomes of interest, the missing values are presented in S1 Table. Missing data on demographics are reported in Table 1. All P values are two-tailed with values of less than 0.05 were considered significant when evaluating the results presented. Analyses were performed in SAS V9.4 (Cary, NC: SAS Institute Inc.).

**Table 1:** Participant characteristics—All patients & clinicians by arm.

	**Arm**			
**Patient Characteristics (baseline and post baseline)**	**ICAN** (N = 832)	**Standard Care** (N = 901)	**Total** (N = 1733)	**P-value**
**Age**: Mean (SD)	62 (15)	60 (15)	61 (15)	0.07^1^
Missing	5	3	8	
**Gender**, n (%)				0.13^2^
Male	354 (42.7%)	352 (39.1%)	706 (40.8%)	
Female	476 (57.3%)	548 (60.9%)	1024 (59.2%)	
Missing	2	1	3	
**Ethnicity**, n (%)				0.14^2^
Hispanic or Latino	19 (2.4%)	12 (1.4%)	31 (1.9%)	
Not Hispanic or Latino	768 (97.6%)	833 (98.6%)	1601 (98.1%)	
Missing	45	56	101	
**Race**, n (%)				< .0001^3^
American Indian or Alaska Native	12 (1.5%)	7 (0.8%)	19 (1.1%)	
Asian	4 (0.5%)	0 (0.0%)	4 (0.2%)	
Black or African American	94 (11.4%)	167 (18.5%)	261 (15.1%)	
More than one race	26 (3.1%)	17 (1.9%)	43 (2.5%)	
White	690 (83.5%)	710 (78.8%)	1400 (81.1%)	
Missing	6	0	6	
**Marital Status**, n (%)				0.27^2^
Married	465 (56.2%)	479 (53.5%)	944 (54.8%)	
Divorced	125 (15.1%)	140 (15.6%)	265 (15.4%)	
Widowed	122 (14.7%)	118 (13.2%)	240 (13.9%)	
Separated	20 (2.4%)	26 (2.9%)	46 (2.7%)	
Never been married	78 (9.4%)	115 (12.8%)	193 (11.2%)	
Member of an unmarried couple	18 (2.2%)	17 (1.9%)	35 (2.0%)	
Missing	4	6	10	
**Employment**, n (%)				0.04^2^
Employed for wages	221 (28.0%)	249 (28.9%)	470 (28.4%)	
Homemaker	44 (5.6%)	32 (3.7%)	76 (4.6%)	
Multiple sources	68 (8.6%)	81 (9.4%)	149 (9.0%)	
Out of work for less than 1 year	3 (0.4%)	14 (1.6%)	17 (1.0%)	
Out of work for more than 1 year	17 (2.2%)	17 (2.0%)	34 (2.1%)	
Retired	286 (36.2%)	333 (38.6%)	619 (37.4%)	
Student	4 (0.5%)	8 (0.9%)	12 (0.7%)	
Unable to work	147 (18.6%)	129 (14.9%)	276 (16.7%)	
Missing	42	38	80	
**Current annual household income**, n (%)				0.11^2^
Less than $20,000	280 (38.7%)	287 (36.8%)	567 (37.7%)	
$20,000 to $34,999	155 (21.4%)	166 (21.3%)	321 (21.4%)	
$35,000 to $49,999	101 (14.0%)	99 (12.7%)	200 (13.3%)	
$50,000 to $74,999	93 (12.9%)	88 (11.3%)	181 (12.0%)	
$75,000 or greater	94 (13.0%)	140 (17.9%)	234 (15.6%)	
Missing	109	121	230	
**Ongoing health conditions**: Mean (SD)	2.6 (1.77)	2.7 (1.77)	2.7 (1.77)	0.28^1^
Missing	34	35	69	
**Survey**, n (%)				0.05^2^
Patient In-Person Survey	612 (73.6%)	624 (69.3%)	1236 (71.3%)	
Patient Postal Survey	220 (26.4%)	277 (30.7%)	497 (28.7%)	
**Clinicians at baseline**	**ICAN** **(N = 37)**	**Standard Care** **(N = 61)**	**Total** **(N = 98)**	**P-value**
**Age**: Mean (SD)	43.9 (12.65)	41.0 (11.96)	42.1 (12.24)	0.25^1^
**Gender**, n (%)				0.045^3^
Male	2 (5.6%)	14 (23.0%)	16 (16.5%)	
Female	34 (94.4%)	47 (77.0%)	81 (83.5%)	
Missing	1	0	1	
**Type of Clinician**, n (%)				0.09^3^
Physician	4 (10.8%)	16 (26.2%)	20 (20.4%)	
Nurse	19 (51.4%)	30 (49.2%)	49 (50.0%)	
Physician Assistant	2 (5.4%)	0 (0.0%)	2 (2.0%)	
Social Worker	1 (2.7%)	0 (0.0%)	1 (1.0%)	
Health Coach	0 (0.0%)	1 (1.6%)	1 (1.0%)	
Other	11 (29.7%)	14 (23.0%)	25 (25.5%)	
**Years in Practice**, n (%)				0.12^3^
0 to 2 years	4 (11.1%)	9 (14.8%)	13 (13.4%)	
>2 to 5 years	7 (19.4%)	6 (9.8%)	13 (13.4%)	
>5 to 10 years	5 (13.9%)	10 (16.4%)	15 (15.5%)	
>10 to 15 years	2 (5.6%)	14 (23.0%)	16 (16.5%)	
>15 years	18 (50.0%)	22 (36.1%)	40 (41.2%)	
Missing	1	0	1	
**Percent of patient panel with chronic conditions**, n (%)				0.32^3^
0% to 25%	1 (2.8%)	2 (3.4%)	3 (3.2%)	
>25% to 50%	4 (11.1%)	7 (12.1%)	11 (11.7%)	
>50% to 75%	19 (52.8%)	20 (34.5%)	39 (41.5%)	
>75% to 100%	12 (33.3%)	29 (50.0%)	41 (43.6%)	
Missing	1	3	4	

^1^Kruskal-Wallis p-value;

^2^Chi-Square p-value;

^3^Fisher Exact p-value;

### Qualitative data

#### Clinician focus groups and interviews

During follow-up visits to intervention sites, we conducted 1-hour focus groups or individual interviews with all consented clinicians to understand their experience in using ICAN. The interview guide was informed by the Normalization Processes Theory [25] (Guide, S2 Appendix).

#### Patient interviews

Similarly, we conducted interviews with patients immediately following their clinical visits using ICAN, using convenience sampling. When patients were consented to participate in the study, they were asked if they were willing to also complete an interview. Patients filling surveys were no longer asked about interviews after we achieved nine interviews at the site. Interviews lasted between 10 and 40 minutes, yielded rich data to achieve saturation (Guide, S3 Appendix).

#### Intervention fidelity data collected

We sought to capture the degree of implementation of the ICAN Discussion Aid during the intervention period with two mechanisms. First, during follow-up visits to all ICAN sites, we conducted chart reviews of patients who completed baseline surveys to determine if they had received ICAN during the implementation period. Because medical records could not be universally configured across all four sites to flag ICAN documentation, we reviewed records with a list of key words that we deemed indicative of ICAN-confirmed or ICAN-concordant visits (S4 Appendix). Additionally, we sought to collect one to two audio- or video-recorded visits per enrolled clinician during follow-up site visits. These were convenience sampled; when a patient agreed to participate, they were asked if they would be willing to allow video or audio recording. Once an enrolled clinician had two recordings, we no longer asked their patients for recordings during the consenting process. Some clinicians allowed their full visits to be recorded, while others chose to turn off the camera after the aid was used. Videos were reviewed with an *a priori* checklist to determine the extent to which the ICAN tool was used as intended.

### Qualitative data analysis

We used an inductive content analysis approach for qualitative data [26]. After reading the transcripts for familiarity, three coders (KRB, PO, AT) first coded three transcripts with line-by-line coding to develop the first draft of a codebook. Subsequently, the team coded transcripts in triplicate using the codebook to calibrate coding and note newly emerging codes. The team met weekly during this process and determined the codebook comprehensive and calibration established after seven transcripts. Then, PO and AT continued to code the remaining 29 transcripts in duplicate. Focus group and clinician interview transcripts required a separate codebook; in addition to inductive codes, deductive codes based on Normalization Process Theory (NPT) were also added [25]. NPT covers four domains required to normalize interventions into routine practice: *coherence*, *cognitive participation*, *collective action*, *and reflexive monitoring* [25, 27]. Briefly, *coherence* describes sense-making work participants in the intervention must do for implementation. *Cognitive participation* describes participants’ engagement with the intervention and implementation process. *Collective action* refers to actions taken to enable the implementation to occur, and *reflexive monitoring* refers to evaluation of the interventions’ value after implementation. Because of the small number of clinician transcripts, all were coded in triplicate.

### Mixed methods triangulation

We followed an embedded mixed methods design, in which the quantitative data are the primary findings and the qualitative data are used to provide greater insight to the quantitative findings [28]. We analyzed quantitative and qualitative data separately and in parallel, and then merged the findings from each for this report [28].

### Patient and public involvement

The Knowledge and Evaluation Research Unit Patient Advisory Group participated in the design of the ICAN Discussion Aid, ensuring its relevance to patients living with chronic conditions and its ease of use. They were not consulted for the research design of this trial.

## Results

### Sample descriptions

**Fig 2:**
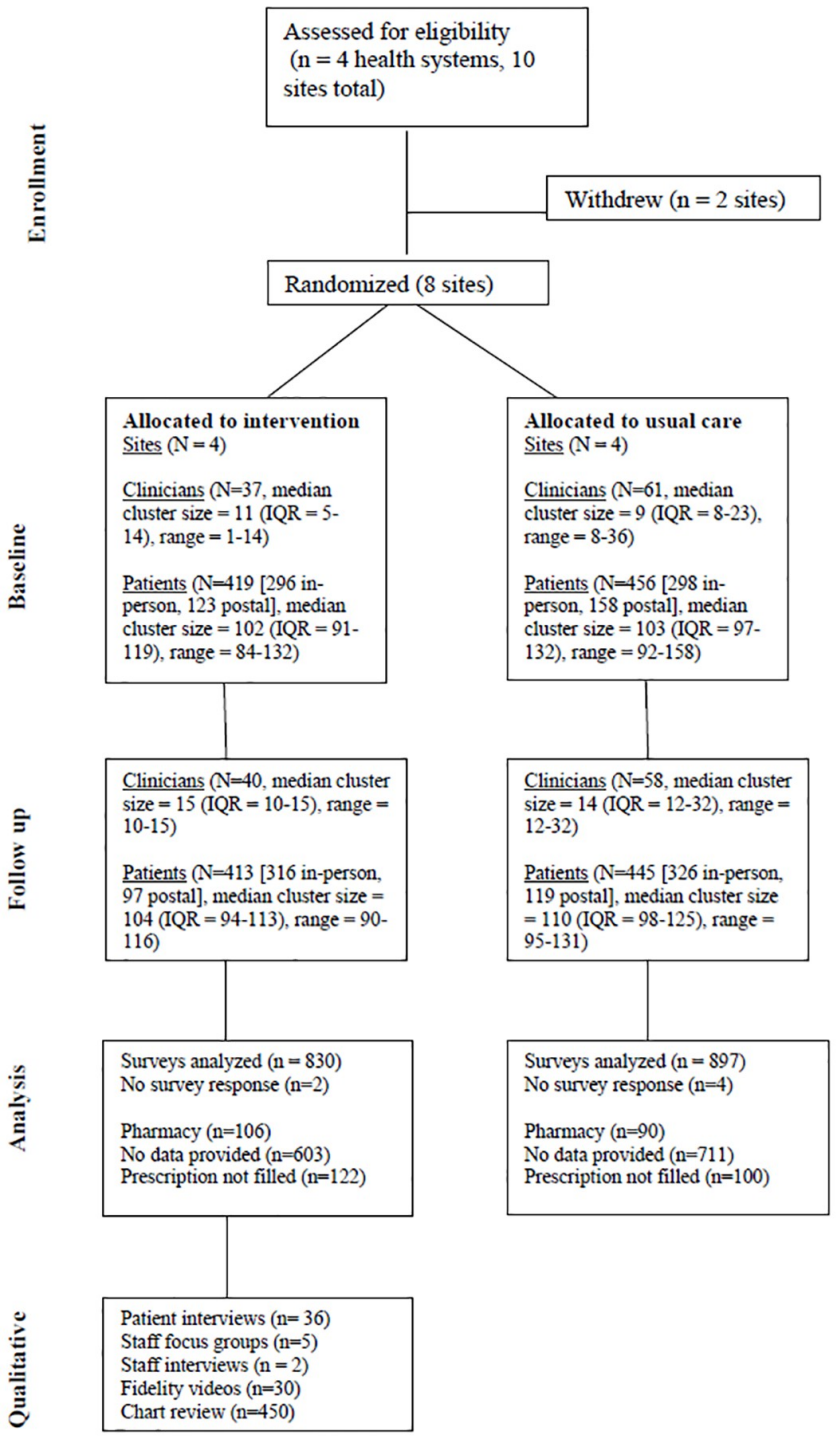
Consort diagram.

Fig 2 depicts the study flow. Table 1 describes the patient and clinician participants in both arms, with analyses to determine if there were significant differences between arms across demographic characteristics. For patient participants, statistically significant differences were seen at baseline between intervention and control sites in race, employment, and survey completion. Among clinician participants, there were statistically significant differences in gender between arms. Patient baseline and follow-up cohorts in each arm were similar, except that the baseline cohort at intervention sites had slightly more ongoing health conditions than the follow-up cohort (mean 2.8 (SD 1.76) vs 2.5 (SD 1.78), p < .05).

### Quantitative results

Tables 2 and 3 report the results from hierarchical generalized linear model for patient reported outcomes and the hierarchical generalized linear model difference-in-difference analyses, respectively. We found no significant differences between arms on any measure in the random effects model. Unadjusted difference-in-difference analyses showed statistically significant improvement Care Consultation Measure subscales of personal relationships [-0.70 (0.34), p = 0.04, lower is better] and clinician interest in the problem’s effect on life [-0.50 (0.24), p = 0.04, lower is better]; self-efficacy to manage chronic disease [0.59 (0.27) p = 0.03, higher is better]; confidence in discussing their condition with their clinician [0.28 (0.14) p = 0.05, higher is better]. In adjusted models, only self-efficacy to manage chronic disease [0.61 (0.27), p = 0.02, higher is better] and confidence in discussing their condition with their clinician [0.31 (0.14), p = 0.03, higher is better] remained significant.

**Table 2:** Mean difference from hierarchical generalized linear model for patient reported outcomes.

Measure	Estimate (95% CI)^a^	P-value
*Patient In Person Survey*		
Care consultation measure (overall)	-0.25 (-3.79, 3.29)	0.89
Communication and partnership	-0.08 (-2.13, 1.97)	0.94
Personal relationship	-0.32 (-1.11, 0.46)	0.46
Health promotion	0.11 (-0.16, 0.38)	0.44
Positive and clear approach to problem	-0.02 (-0.35, 0.31)	0.91
Interest in effect on life	0.06 (-0.30, 0.42)	0.74
Treatment burden	-1.88 (-5.92, 2.17)	0.36
Self-efficacy to manage disease	0.08 (-1.57, 1.73)	0.92
Confidence in discussing with clinician	-0.21 (-0.64, 0.22)	0.33
*Patient Postal Survey*		
Self-rated health	0.25 (-0.21, 0.70)	0.28
PACIC (overall)	-0.04 (-0.34, 0.26)	0.79
Patient activation	-0.15 (-0.4, 0.13)	0.30
Delivery system design/decision support	-0.01 (-0.36, 0.34)	0.96
Goal setting	-0.02 (-0.37, 0.32)	0.89
Problem solving/contexture counselling	-0.05 (-0.45, 0.35)	0.81
Follow-up/coordination	-0.08 (-0.39, 0.23)	0.61
Illness intrusiveness scale (overall)	0.30 (-0.40, 1.00)	0.40
Physical well-being and diet	0.18 (-0.41, 0.76)	0.56
Work and finances	0.23 (-0.66, 1.12)	0.61
Marital, sexual and family relations	0.42 (-0.17, 0.99)	0.16
Recreation and social relations	0.46 (-0.31, 1.23)	0.24
Other aspects of life	0.29 (-0.52, 1.10)	0.49

^a^—Mean difference of ICAN–Standard Care from Hierarchical Generalized Linear effect model with random effect of site, adjusted by fixed effects of arm, timepoint, the interaction of arm and timepoint, age, sex, race, ethnicity, and number of comorbid conditions

**Table 3:** Difference-in-difference analyses of patient-reported outcomes.

Measure	All Healthcare Systems							
	Unadjusted Estimate (SE)^a^				Adjusted Estimate (SE)^d^			
	Standard Care^b^	ICAN^b^	D-I-D^c^	P-value	Standard Care^b^	ICAN^b^	D-I-D^c^	P-value
*Patient In Person Survey*								
Care consultation measure (overall)	-0.32 (1.36)	-3.57 (1.39)	-3.24 (1.94)	0.09	-0.42 (1.43)	-3.47 (1.43)	-3.05 (2.02)	0.13
Communication and partnership	-0.54 (0.76)	-1.53 (0.76)	-1.00 (1.08)	0.35	-0.46 (0.80)	-1.45 (0.80)	-0.98 (1.12)	0.38
Personal relationship	0.11 (0.24)	-0.59 (0.24)	**-0.70 (0.34)**	**0.04**	0.05 (0.25)	-0.61 (0.25)	-0.66 (0.35)	0.07
Health promotion	0.13 (0.17)	-0.31 (0.17)	-0.44 (0.24)	0.06	-0.11 (0.17)	-0.25 (0.17)	-0.36 (0.25)	0.14
Positive and clear approach to problem	0.09 (0.23)	-0.52 (0.23)	-0.61 (0.33)	0.06	0.06 (0.24)	-0.55 (0.24)	-0.62 (0.34)	0.07
Interest in effect on life	-0.13 (0.17)	-0.63 (0.17)	**-0.50 (0.24)**	**0.04**	-0.18 (0.18)	-0.61 (0.18)	-0.43 (0.26)	0.09
Treatment burden	-1.41 (1.44)	-2.64 (1.45)	-1.23 (2.04)	0.55	-1.58 (1.43)	-1.89 (1.43)	-0.32 (2.0)	0.88
Self-efficacy to manage disease	-0.23 (0.19)	0.36 (0.19)	**0.59 (0.27)**	**0.03**	-0.37 (0.19)	0.24 (0.19)	**0.61 (0.27)**	**0.02**
Confidence in discussing with clinician	-0.15 (0.10)	0.13 (0.10)	**0.28 (0.14)**	**0.05**	-0.18 (0.10)	0.13 (0.10)	**0.31 (0.14)**	**0.03**
*Patient Postal Survey*								
Self-rated health	0.06 (0.12)	0.14 (0.14)	0.78 (0.18)	0.67	0.06 (0.12)	0.07 (0.14)	0.01 (0.19)	0.96
PACIC (overall)	-0.13 (0.14)	-0.05 (0.16)	0.08 (0.21)	0.69	-0.13 (0.14)	-0.02 (0.16)	0.12 (0.22)	0.59
Patient activation	0.02 (0.17)	-0.14 (0.19)	-0.16 (0.26)	0.53	-0.02 (0.18)	-0.11 (0.20)	-0.10 (0.27)	0.72
Delivery system design/decision support	-0.07 (0.15)	-0.13 (0.17)	-0.06 (0.22)	0.79	-0.10 (0.15)	-0.10 (0.17)	-0.00 (0.23)	0.99
Goal setting	-0.02 (0.15)	-0.14 (0.17)	-0.13 (0.23)	0.58	0.01 (0.16)	-0.08 (0.18)	-0.09 (0.24)	0.71
Problem solving/contexture counselling	-0.23 (0.17)	0.17 (0.19)	0.49 (0.26)	0.12	-0.25 (0.18)	0.14 (0.20)	0.39 (0.27)	0.15
Follow-up/coordination	-0.16 (0.15)	-0.10 (0.17)	0.06 (0.23)	0.78	-0.17 (0.15)	-0.02 (0.18)	0.15 (0.23)	0.52
Illness intrusiveness scale (overall)	0.34 (0.19)	0.60 (0.22)	0.25 (0.29)	0.38	0.29 (0.20)	0.65 (0.23)	0.36 (0.31)	0.23
Physical well-being and diet	0.43 (0.23)	0.50 (0.26)	0.07 (0.34)	0.85	0.42 (0.23)	0.52 (0.26)	0.10 (0.35)	0.77
Work and finances	0.28 (0.24)	0.74 (0.27)	0.45 (0.36)	0.21	0.28 (0.25)	0.76 (0.28)	0.48 (0.38)	0.21
Marital, sexual and family relations	0.30 (0.23)	0.43 (0.26)	0.13 (0.35)	0.72	0.26 (0.25)	0.54 (0.29)	0.29 (0.38)	0.45
Recreation and social relations	0.40 (0.21)	0.68 (0.23)	0.28 (0.31)	0.37	0.31 (0.22)	0.73 (0.25)	0.42 (0.33)	0.21
Other aspects of life	0.29 (0.22)	0.69 (0.25)	0.40 (0.33)	0.23	0.29 (0.23)	0.77 (0.27)	0.48 (0.35)	0.18

^a^. Unadjusted estimates, clustered by site

^b^. Least Squares Means differences between time points within arms

^c^. Difference in difference analysis comparing post to pre intervention between arms (ICAN vs. Standard Care)

^d^. Adjusted for fixed effects of age, sex, race, ethnicity and number of comorbid conditions, and random effect of site

^e^. Consultation care measure, lower = better

Adherence measures were better at intervention sites at both baseline and at follow-up (S2 Table). Health professional outcomes (S3 Table), indicated significant improvement in clinician Assessment of Chronic Illness Care (ACIC) scores at ICAN sites compared to usual care. No aspects of relational coordination were significantly different.

### Qualitative results

#### Staff views on ICAN

There were significant implementation challenges at three of the four ICAN sites. These included a major change in clinic leadership with large staff turnover, community challenges from the opioid crisis, a new electronic health record, and clinic closure (Table 4, Quotes 1a, 1b). The one site that did not experience implementation challenges in the broader context, reviewed the tool positively (1c).

**Table 4:** Qualitative supporting quotes.

Staff Views on ICAN	
Quote ID	
1a	*“One thing that really concerns me that we’re lacking in this area that you don’t see in the city or in a suburb*, *we’re seeing all this drug abuse*. *You hear about how big this area and [other state] is in drugs*. *We’re just almost zilch as far as referral to drug addiction specialists*. *I mean we’re overwhelmed*. *They don’t have enough facilities*. *There’s none locally*. *… [The nearest one is] about an hour away basically*. *But they only keep them like three days*. *… We have no way to treat these patients*.*” Clinician 1*, *Site 1*
1b	*“We really transitioned a lot for our patients though between portal and iPads when they come in the door and Epic [electronic health record]*. *There’s been a lot of change for them*. *So that was just something that added more for them*, *and I think they’re still adjusting too*.*” Clinician 2*, *Site 3*
1c	*“Yeah*, *you know*, *once you start to know your patients*, *you know who’s very vocal and who comes with their list and who usually answers yes*, *no*, *very short answers*. *And in that kind of patient*, *I am more likely to use it*. *Some people may not want to verbalize their thoughts so they might feel more comfortable writing them down*. *… I know if they come in for diabetes*, *high blood pressure*, *and so forth*, *I already know in my mind what I’m going to do for those*. *And then if I see it written down on the tool*, *oh there’s a couple things written down here*, *then I*, *uh I’ll allot my time a little better*.*” Clinician 3*, *Site 4*
1d	*“I feel like in the very beginning you know*, *you just kinda open your eyes to the whole non-compliance issue*. *You know*, *we are just quick to write somebody off that they’re non-compliant*, *but why are they*? *We learned*, *you know*, *maybe there’s other barriers–other factors–that are making them that way*, *not just a conscious choice to not take their medicine and not do what they’re supposed to do*.*” Clinician 4*, *Site 2*
1e	*“We are trying to fix health problems*, *but the patients’ other problems which is contributed to their health problems*, *we don’t have time to assess*. *That’s just the way it works*. *If a patient’s telling me that she’s got depression and anxiety*, *and it’s over because she doesn’t have money to live on*, *and then she gets a welfare check or some help*. *And then she doesn’t come back for depression ‘cause she’s like oh*, *I don’t have depression no more*, *I’ve got some money*. *It’s just a real–it’s a social issue*. *A lot of them*.*” Clinician 5*, *Site 1*
1f	*“So I think when patients come in and they’re having a lot of barriers and they’ve got a lot of problems we are not able to meet–or help the needs–because of something that would have been checked on the tool–we know it before that tool is filled out*. *Um*, *so I think that’s a big thing that we’re learning from–we don’t need the tool to help us do what we need to do to make patients able to meet their healthcare needs*.*” Clinician6*, *Site 2*
Patient Views on ICAN	
2a	*“Normal doctor’s appointments*, *you don’t think to talk about your work*, *except I do ‘cause they’re all smokers*. *But most people would note*. *Like my husband*. *He wouldn’t think about that*. *He loves his job*, *but he works underground in the mines*. *A lot of things go on down there*. *Um*, *stuff he needs to tell*, *even like that light sensitivity of his eyes*.*” -Patient 1*, *Site 1*
2b	*“Yes it did [bring out conversation]*. *We would have just gone through the prep thing*, *you know*. *[Nurse] is one of the people that preps you before the doctor sees you*. *Although I have always liked her*, *I would have never found out about our parallels in life issues if I hadn’t gone through that*.*” Patient 2*, *Site 2*
2c	*“But uh*, *I do think that… it’s really a concern to lose a facility like this to see how much business there was done*, *uh*, *at one time*. *I think there were six doctors working out of this facility*, *and uh*, *come by the end of the year there’ll be no full-time doctors and that’s what hurts*.*” Patient 3*, *Site 3*
2d	*“I don’t know*, *just another survey*. *I guess if I had concerns about healthcare and what’s going on*, *I would–it would be uh*, *more pertinent to me*. *… I come here and things go well*, *and I have no problems so it makes it easy*.*” Patient 4*, *Site 3*
2e	*“I hate to say no [it wasn’t helpful]*. *I don’t think it made much difference*. *Well*, *I was gonna talk about those things*. *I put one down–one thing on here where it says ‘being active*.*’ I wrote in ‘I always feel tired’ and we didn’t really talk about that*.*” Patient 5*, *Site 2*
2f	*“I don’t like ‘em [Lasix blood pressure pill]*. *They run you to death [patient’s expression for needing to run to the bathroom often]*. *… I am supposed to take ‘em all [medications]*, *but I don’t always take my Lasix*. *… Usually if I am on the road*, *I don’t take ‘em*.*” Patient 6*, *Site 1*

Using NPT, across all sites there was a coherence of understanding the reason for the aid’s use. Specifically, staff found the training which reframed “non-compliance” more empathically as impactful (1d). Breakdowns occurred when cognitive participation and collective action were required because staff felt they could not “fix” the psychosocial issues arising from ICAN use (1e) or they perceived their usual conversations without the tool covered the same content (1f). However, patients’ views, for the most part, did not echo this sentiment. Finally, in reflexive monitoring, staff noted a lack of ICAN workflow in their EHR and the perception that the visits took longer with ICAN.

#### Patient views on ICAN

Most patients found the use of ICAN to be a positive experience. Patients neutral to ICAN’s use stated that it didn’t add anything to their visit that day, but it would be helpful for others or in difference circumstances (2b, 2c). Patients with negative reactions to ICAN use mostly came from the site that was to be closing shortly (3c). At this site, patients described ICAN as a “survey”, whereas at other sites, patients’ language mostly reflected using it for discussion or personal reflection (3d). It is unclear if this was due to a difference in patient perception of the tool’s usefulness or that site more commonly deployed surveys compared to others. Specific changes to ICAN requested by patients, included adding a “finances” line in the areas of life section, pre-filled topics in the treatment section, and emoticons to represent satisfaction/help versus burden.

#### Fidelity to ICAN use and training

In 19 of 36 interviews, patients did not recall their clinician looking at their ICAN, despite interviews occurring immediately after the visit. Visits recorded showed that 22 of 30 clinicians did look at ICAN during the visit, although use was not as intended. Patients sometimes expressed disappointment that their clinician had not reviewed their answers (2e). In some situations, non-adherence to medication was missed due to not using the tool (2f). Chart review of patients recruited in the baseline cohort at the intervention sites showed that 19% of patient charts had direct or indirect evidence of ICAN use during the implementation period.

## Discussion

### Summary of findings

In this pragmatic mixed-methods cluster-randomized trial, we found ICAN did not have a clear, consistent, and significant effect on patient-reported measures of care or on adherence to treatment. The tool was used infrequently and often not as intended. Yet, when both patient and clinician were invested in using ICAN, some useful conversations emerged.

### Relationship to other literature

The ICAN Discussion Aid is one of many interventions for use with patients living with multimorbidity, yet there are few conversation or decision aids designed for eliciting priorities with this population, as called for by the Academy of Medical Sciences in 2018 [29, 30]. ICAN is also the first intervention designed using Minimally Disruptive Medicine’s focus on treatment workload and patient capacity [4, 31]. Early pilot work showed ICAN’s impact on clinical conversation topics [10], but further improvements in patient-reported outcomes within this study were not observed. Given our qualitative findings, it remains possible that this lack of effect is either true or an artifact of the limited use of ICAN at intervention sites. These results should give pause to healthcare systems considering the implementation of ICAN.

ICAN was designed for sustainable use within regular clinic workflows. Other interventions with similar aims but requiring additional staff have been successful. The Patient Journeys Record System used lay care guides to have semi-structured conversations with patients living with chronic illness at high risk for hospitalization, and it was found to be effective in reducing unplanned hospitalizations in a randomized trial [32]. The Patient Priorities Care for elderly patients with multimorbidity trained clinicians to elicit patient priorities and fed forward this information to plans of care leading to improvements in deprescribing and burden of treatment [33]. Our qualitative findings would suggest that for ICAN to be effective in usual care, greater investments in training and implementation may be necessary.

Beyond additional staff, ICAN may require broader cultural changes within healthcare systems. ICAN is designed to highlight the work that patients do to care for their conditions and the capacity required to do so with the underlying premise that health professionals using the tool endorse these issues as important to patients’ care and quality of life. Without cultural investments at institutional level, one-off ICAN implementation by individual clinicians may fall short, as we saw in many instances.

### Strengths and limitations

Our research is not without limitations. First, we needed to enroll different patient cohorts at baseline and follow up required because of the data collection at remote sites, many without any research experience or support. However, this also gives us confidence in the pragmatic nature of the trial, and therefore, the greater likelihood of the generalizability of our findings. Second, due to unforeseen circumstances, we also had to replace two ICAN sites after randomization, and finally, the use of ICAN in a paper-based format appears to have discouraged use. Since study completion, we have integrated ICAN into the Epic EHR.

### Implications for research and practice

We followed a traditional pathway from intervention development, to pilot study, and then a larger trial. However, it is clear from our mixed methods approach that additional qualitative and implementation science research should have preceded the effectiveness trial, and we would encourage similar research to proceed with this work in advance. One potential option is hybrid implementation-effectiveness designs [34], in alignment with more nuanced depictions of the dissemination and implementation sciences [35].

A sanguine view of our results would indicate that our prior research that ICAN can change conversations [10] aligns with the present qualitative findings–connection and partnership can emerge when patient and clinician use ICAN to craft meaningful conversations. With leadership and participant buy-in, NPT would predict that well supported clinic staff could work together to integrate ICAN within existing workflows. In follow-up discussions with the clinical site that reported the highest levels of implementation reported that early successes in using the tool, such as a particularly meaningful patient visit during the training period, were impactful in spearheading enthusiastic use. Healthcare professionals and system leaders who wish to test this prediction in their practice, can freely access ICAN and an implementation toolkit at https://carethatfits.org/my-life-my-healthcare/, informed by stakeholders who participated in this trial and by ongoing implementation work [36].

## Conclusion

In this large, multi-site cluster randomized trial of the ICAN Discussion Aid, we found no significant improvements across most quantitative measures including patient-clinician communication and treatment burden. Qualitative data suggest the tool was used infrequently and often not as intended. Yet, when both patient and clinician were invested in using ICAN, human connection and tailored care emerged. Subsequent work will focus on interventions to promote appropriate and high-fidelity use of ICAN in practice to enable estimations of its value in real-world practices employing hybrid effectiveness-implementation trials.

## Supporting information

S1 Table Unadjusted quantitative results with missing counts. (DOCX)

S2 Table Analyses of medication adherence. (DOCX)

S3 Table Difference-in-differences analyses of health professional-reported outcomes. (DOCX)

S1 Appendix Survey scoring. (PDF)

S2 Appendix Clinician focus group/interview guide. (PDF)

S3 Appendix Patient interview guide. (PDF)

S4 Appendix EMR review guide. (PDF)
