# Supplementary material for: Care for patients living with chronic conditions using the ICAN Discussion Aid: A mixed methods cluster-randomized trial
Source: PLoS One. 2024 Dec 4;19(12):e0314605. doi: 10.1371/journal.pone.0314605 (PMC11616879; doi:10.1371/journal.pone.0314605)
Supplement: S1 Table — (DOCX) [file pone.0314605.s001.docx]

**Supplement Table 1:** **Unadjusted Quantitative Results with Missing Counts**

***Patient In-Person Survey - Descriptive Statistics***

|  | | | | | | | |
| --- | --- | --- | --- | --- | --- | --- | --- |
|  | Arm | | | | | | |
|  | ICAN | | |  | Standard Care | | |
|  | Timepoint | |  |  | Timepoint | |  |
|  | Baseline (N=296) | Post-Baseline (N=316) | Total (N=612) |  | Baseline (N=298) | Post-Baseline (N=326) | Total (N=624) |
| **Communicating with your physician** |  |  |  |  |  |  |  |
| Mean (SD) | 2.5 (1.33) | 2.6 (1.23) | 2.6 (1.28) |  | 2.9 (1.25) | 2.7 (1.18) | 2.8 (1.22) |
| Missing | 12 | 4 | 16 |  | 9 | 10 | 19 |
| **Self-efficacy in managing chronic disease** |  |  |  |  |  |  |  |
| Mean (SD) | 6.2 (2.55) | 6.5 (2.55) | 6.4 (2.55) |  | 6.4 (2.49) | 6.2 (2.58) | 6.3 (2.54) |
| Missing | 14 | 11 | 25 |  | 10 | 16 | 26 |
| **CCM-Communication and partnership** |  |  |  |  |  |  |  |
| Mean (SD) | 20.1 (8.98) | 18.5 (9.33) | 19.3 (9.18) |  | 19.7 (9.40) | 19.1 (9.50) | 19.4 (9.45) |
| Missing | 4 | 22 | 26 |  | 9 | 15 | 24 |
| **CCM-Personal relationship** |  |  |  |  |  |  |  |
| Mean (SD) | 6.0 (2.88) | 5.4 (2.78) | 5.7 (2.85) |  | 6.0 (2.93) | 6.1 (3.13) | 6.1 (3.03) |
| Missing | 4 | 22 | 26 |  | 9 | 15 | 24 |
| **CCM-Health promotion** |  |  |  |  |  |  |  |
| Mean (SD) | 4.2 (2.03) | 3.9 (2.04) | 4.1 (2.04) |  | 3.9 (1.97) | 4.0 (2.06) | 4.0 (2.02) |
| Missing | 4 | 22 | 26 |  | 9 | 15 | 24 |
| **CCM-Positive and clear approach to problem** |  |  |  |  |  |  |  |
| Mean (SD) | 6.2 (2.77) | 5.7 (2.75) | 5.9 (2.77) |  | 5.9 (2.73) | 6.0 (2.92) | 5.9 (2.83) |
| Missing | 4 | 22 | 26 |  | 9 | 15 | 24 |
| **CCM-Interest in effect on life** |  |  |  |  |  |  |  |
| Mean (SD) | 4.5 (2.13) | 3.9 (2.04) | 4.2 (2.10) |  | 4.2 (2.14) | 4.1 (2.12) | 4.1 (2.13) |
| Missing | 4 | 22 | 26 |  | 9 | 15 | 24 |
| **CCM-Total** |  |  |  |  |  |  |  |
| Mean (SD) | 41.0 (16.49) | 37.4 (16.51) | 39.2 (16.58) |  | 39.6 (16.80) | 39.3 (17.31) | 39.5 (17.05) |
| Missing | 4 | 22 | 26 |  | 9 | 15 | 24 |
| **Treatment burden questionnaire** |  |  |  |  |  |  |  |
| Mean (SD) | 16.1 (17.50) | 13.4 (16.23) | 14.8 (16.92) |  | 17.4 (19.23) | 16.0 (17.99) | 16.7 (18.61) |
| Missing | 2 | 19 | 21 |  | 4 | 20 | 24 |
|  | | | | | | | |

***Patient Postal Survey - Descriptive Statistics***

|  | | | | | | | |
| --- | --- | --- | --- | --- | --- | --- | --- |
|  | Arm | | | | | | |
|  | ICAN | | |  | Standard Care | | |
|  | Timepoint | |  |  | Timepoint | |  |
|  | Baseline (N=123) | Post-Baseline (N=97) | Total (N=220) |  | Baseline (N=158) | Post-Baseline (N=119) | Total (N=277) |
| **Self-rated health** |  |  |  |  |  |  |  |
| Mean (SD) | 3.3 (1.03) | 3.5 (0.95) | 3.4 (0.99) |  | 3.2 (1.01) | 3.2 (1.07) | 3.2 (1.03) |
| Missing | 4 | 0 | 4 |  | 5 | 1 | 6 |
| **PACIC-Patient activation** |  |  |  |  |  |  |  |
| Mean (SD) | 3.2 (1.41) | 3.1 (1.31) | 3.2 (1.37) |  | 3.3 (1.35) | 3.3 (1.35) | 3.3 (1.35) |
| Missing | 10 | 8 | 18 |  | 12 | 8 | 20 |
| **PACIC-Delivery system design** |  |  |  |  |  |  |  |
| Mean (SD) | 3.5 (1.17) | 3.4 (1.20) | 3.5 (1.18) |  | 3.5 (1.17) | 3.4 (1.14) | 3.4 (1.16) |
| Missing | 10 | 6 | 16 |  | 12 | 8 | 20 |
| **PACIC-Goal setting** |  |  |  |  |  |  |  |
| Mean (SD) | 3.0 (1.14) | 2.8 (1.25) | 2.9 (1.19) |  | 2.9 (1.25) | 2.9 (1.16) | 2.9 (1.21) |
| Missing | 10 | 7 | 17 |  | 12 | 8 | 20 |
| **PACIC-Problem solving** |  |  |  |  |  |  |  |
| Mean (SD) | 3.1 (1.36) | 3.3 (1.30) | 3.2 (1.34) |  | 3.4 (1.37) | 3.1 (1.35) | 3.3 (1.36) |
| Missing | 9 | 9 | 18 |  | 12 | 8 | 20 |
| **PACIC-Follow-up/coordination** |  |  |  |  |  |  |  |
| Mean (SD) | 2.5 (1.19) | 2.4 (1.18) | 2.4 (1.18) |  | 2.5 (1.23) | 2.4 (1.16) | 2.5 (1.20) |
| Missing | 9 | 9 | 18 |  | 12 | 8 | 20 |
| **PACIC-Total** |  |  |  |  |  |  |  |
| Mean (SD) | 3.0 (1.07) | 3.0 (1.07) | 3.0 (1.07) |  | 3.1 (1.11) | 3.0 (1.08) | 3.0 (1.10) |
| Missing | 12 | 10 | 22 |  | 15 | 8 | 23 |
| **IL-Physical well-being and diet** |  |  |  |  |  |  |  |
| Mean (SD) | 3.5 (1.94) | 4.0 (1.90) | 3.7 (1.94) |  | 3.3 (1.77) | 3.8 (1.96) | 3.5 (1.86) |
| Missing | 1 | 0 | 1 |  | 2 | 0 | 2 |
| **IL-Work and finances** |  |  |  |  |  |  |  |
| Mean (SD) | 3.1 (1.96) | 3.9 (2.00) | 3.4 (2.01) |  | 3.1 (2.04) | 3.4 (2.03) | 3.2 (2.04) |
| Missing | 2 | 1 | 3 |  | 3 | 6 | 9 |
| **IL-Marital, sexual and family relations** |  |  |  |  |  |  |  |
| Mean (SD) | 2.7 (1.92) | 3.2 (2.13) | 2.9 (2.03) |  | 2.4 (1.82) | 2.7 (1.92) | 2.5 (1.87) |
| Missing | 2 | 0 | 2 |  | 1 | 0 | 1 |
| **IL-Recreation and social relations** |  |  |  |  |  |  |  |
| Mean (SD) | 2.9 (1.75) | 3.7 (1.81) | 3.3 (1.81) |  | 2.7 (1.65) | 3.1 (1.79) | 2.9 (1.72) |
| Missing | 2 | 0 | 2 |  | 2 | 1 | 3 |
| **IL-Other aspects of life** |  |  |  |  |  |  |  |
| Mean (SD) | 2.5 (1.85) | 3.2 (2.08) | 2.8 (1.99) |  | 2.4 (1.76) | 2.7 (1.83) | 2.5 (1.79) |
| Missing | 2 | 1 | 3 |  | 1 | 0 | 1 |
| **IL-Total** |  |  |  |  |  |  |  |
| Mean (SD) | 2.9 (1.61) | 3.6 (1.65) | 3.2 (1.66) |  | 2.8 (1.52) | 3.1 (1.67) | 2.9 (1.59) |
| Missing | 4 | 1 | 5 |  | 3 | 6 | 9 |
|  | | | | | | | |

***Clinician Survey - Descriptive Statistics***

|  | | | | | | | |
| --- | --- | --- | --- | --- | --- | --- | --- |
|  | Arm | | | | | | |
|  | ICAN | | |  | Standard Care | | |
|  | Timepoint | |  |  | Timepoint | |  |
|  | Baseline (N=37) | Post-Baseline (N=40) | Total (N=77) |  | Baseline (N=61) | Post-Baseline (N=58) | Total (N=119) |
| **ACIC Overall** |  |  |  |  |  |  |  |
| Mean (SD) | 6.3 (1.77) | 7.4 (1.67) | 6.9 (1.80) |  | 8.0 (1.95) | 7.8 (2.02) | 7.9 (1.98) |
| Missing | 2 | 2 | 4 |  | 7 | 5 | 12 |
| **ACIC-Organization of healthcare delivery system** |  |  |  |  |  |  |  |
| Mean (SD) | 7.1 (1.73) | 7.6 (2.03) | 7.4 (1.90) |  | 8.2 (1.93) | 7.9 (1.87) | 8.1 (1.90) |
| Missing | 1 | 1 | 2 |  | 1 | 4 | 5 |
| **ACIC-Community linkages** |  |  |  |  |  |  |  |
| Mean (SD) | 6.4 (1.95) | 7.5 (1.92) | 7.0 (2.00) |  | 7.3 (2.65) | 7.1 (2.73) | 7.2 (2.68) |
| Missing | 1 | 2 | 3 |  | 1 | 3 | 4 |
| **ACIC-Self-management support** |  |  |  |  |  |  |  |
| Mean (SD) | 6.1 (1.78) | 7.4 (2.17) | 6.7 (2.08) |  | 7.7 (2.23) | 7.7 (2.37) | 7.7 (2.29) |
| Missing | 2 | 2 | 4 |  | 6 | 3 | 9 |
| **ACIC-Decision support** |  |  |  |  |  |  |  |
| Mean (SD) | 6.3 (2.52) | 7.2 (2.05) | 6.8 (2.32) |  | 7.7 (2.46) | 7.4 (2.52) | 7.6 (2.48) |
| Missing | 1 | 2 | 3 |  | 1 | 3 | 4 |
| **ACIC-Delivery system design** |  |  |  |  |  |  |  |
| Mean (SD) | 6.2 (1.89) | 7.3 (1.85) | 6.7 (1.93) |  | 8.0 (2.01) | 8.0 (2.29) | 8.0 (2.14) |
| Missing | 1 | 2 | 3 |  | 1 | 3 | 4 |
| **ACIC-Clinical information systems** |  |  |  |  |  |  |  |
| Mean (SD) | 5.9 (2.29) | 7.2 (1.80) | 6.6 (2.14) |  | 7.7 (2.34) | 7.7 (2.43) | 7.7 (2.37) |
| Missing | 1 | 2 | 3 |  | 2 | 3 | 5 |
| **Relational Coordination (RC) - Overall** |  |  |  |  |  |  |  |
| Mean (SD) | 2.7 (0.84) | 2.8 (0.84) | 2.7 (0.84) |  | 2.3 (1.01) | 2.7 (0.96) | 2.5 (0.99) |
| Missing | 3 | 4 | 7 |  | 5 | 10 | 15 |
| **RC - Frequent** |  |  |  |  |  |  |  |
| Mean (SD) | 3.2 (1.07) | 3.5 (1.16) | 3.3 (1.12) |  | 3.1 (1.08) | 3.4 (1.08) | 3.2 (1.09) |
| Missing | 3 | 2 | 5 |  | 2 | 6 | 8 |
| **RC - Timely** |  |  |  |  |  |  |  |
| Mean (SD) | 2.9 (1.10) | 2.7 (1.14) | 2.8 (1.12) |  | 2.2 (1.14) | 2.4 (1.05) | 2.3 (1.10) |
| Missing | 3 | 2 | 5 |  | 2 | 4 | 6 |
| **RC - Accurate** |  |  |  |  |  |  |  |
| Mean (SD) | 2.7 (1.23) | 2.6 (1.16) | 2.6 (1.19) |  | 2.0 (1.10) | 2.3 (1.04) | 2.2 (1.08) |
| Missing | 2 | 2 | 4 |  | 2 | 3 | 5 |
| **RC – Problem solving** |  |  |  |  |  |  |  |
| Mean (SD) | 2.8 (1.14) | 2.8 (1.04) | 2.8 (1.08) |  | 2.3 (1.23) | 2.6 (1.18) | 2.5 (1.21) |
| Missing | 2 | 2 | 4 |  | 2 | 4 | 6 |
| **RC - Goals** |  |  |  |  |  |  |  |
| Mean (SD) | 2.4 (1.00) | 2.4 (1.19) | 2.4 (1.09) |  | 2.3 (1.13) | 2.3 (1.17) | 2.3 (1.14) |
| Missing | 2 | 2 | 4 |  | 4 | 4 | 8 |
| **RC - Knowledge** |  |  |  |  |  |  |  |
| Mean (SD) | 2.7 (1.13) | 2.8 (0.86) | 2.7 (0.99) |  | 2.5 (1.19) | 2.6 (1.15) | 2.5 (1.17) |
| Missing | 2 | 2 | 4 |  | 2 | 3 | 5 |
|  | | | | | | | |
