# Supplementary material for: Care for patients living with chronic conditions using the ICAN Discussion Aid: A mixed methods cluster-randomized trial
Source: PLoS One. 2024 Dec 4;19(12):e0314605. doi: 10.1371/journal.pone.0314605 (PMC11616879; doi:10.1371/journal.pone.0314605)
Supplement: S2 Table — (DOCX) [file pone.0314605.s002.docx]

After excluding medication classes with less than 5 patients

| **Supplement Table 2:** **Analyses of medication adherence** | | | |
| --- | --- | --- | --- |
|  | ICAN (N=106) | Standard Care  (N=90) | p value |
| **Count of medications prior to encounter** |  |  | 0.24^b^ |
| Mean (SD) | 5.6 (4.9) | 6.5 (5.2) |  |
| **Count of medication classes prior to encounter** |  |  | 0.22^b^ |
| Mean (SD) | 3.1 (2.3) | 3.4 (2.1) |  |
| **Adherence (PDC) prior to encounter** |  |  | 0.01^b^ |
| Mean (95% CI) | 35% (32, 38) | 31% (28, 34) |  |
| **Count of medications post encounter** |  |  | 0.06^b^ |
| Mean (SD) | 5.5 (5.0) | 6.7 (5.2) |  |
| **Count of medication classes post encounter** |  |  | 0.09^b^ |
| Mean (SD) | 3.1 (2.4) | 3.5 (2.1) |  |
| **Adherence (PDC) post encounter** |  |  | 0.01^b^ |
| Mean (95% CI) | 41% (38, 45) | 37% (33, 40) |  |
| **Adjusted adherence (PDC) post encounter ^a^** |  |  | 0.46 |
| Mean (95% CI) | 39% (37, 42) | 38% (36, 41) |  |

Acronym: PDC - Percent Days covered, SD – Standard Deviation, IQR – Interquartile range, CI – Confidence Interval

a - Heirarchical generalized linear model with a random effect of site, and fixed effect of baseline PDC, arm and an indicator if baseline PDC is missing.

b – Kruskal Wallis Test
