# Supplementary material for: Care for patients living with chronic conditions using the ICAN Discussion Aid: A mixed methods cluster-randomized trial
Source: PLoS One. 2024 Dec 4;19(12):e0314605. doi: 10.1371/journal.pone.0314605 (PMC11616879; doi:10.1371/journal.pone.0314605)
Supplement: S3 Table — (DOCX) [file pone.0314605.s003.docx]

| **Supplement 3: Difference-in-differences analyses of health professional-reported outcomes** | | | | | | | | |
| --- | --- | --- | --- | --- | --- | --- | --- | --- |
| **Measure** | **All Healthcare Systems** | | | | | | | |
|  | Unadjusted Estimate (SE)^a^ | | | | Adjusted Estimate (SE)^d^ | | | |
|  | Standard Care^b^ | ICAN^b^ | D-I-D^c^ | P-value | Standard Care^b^ | ICAN^b^ | D-I-D^c^ | P-value |
| *Clinician Survey* |  |  |  |  |  |  |  |  |
| **ACIC Overall** | -0.14 (0.35) | 1.08 (0.42) | **1.22 (0.55)** | **0.03** | -0.22 (0.33) | 1.20 (0.40) | **1.42 (0.52)** | **0.007** |
| Organization | -0.10 (0.35) | 0.54 (0.42) | 0.64 (0.55) | 0.25 | -0.19 (0.34) | 0.44 (0.41) | 0.63 (0.53) | 0.24 |
| Community Linkages | -0.03 (0.43) | 1.07 (0.52) | 1.11 (0.68) | 0.10 | -0.28 (0.42) | 1.17 (0.51) | **1.45 (0.66)** | **0.03** |
| Self-Management | -0.01 (0.40) | 1.28 (0.48) | **1.29 (0.63)** | **0.04** | -0.09 (0.38) | 1.48 (0.46) | **1.58 (0.59)** | **0.009** |
| Decision Support | -0.21 (0.44) | 0.90 (0.54) | 1.11 (0.70) | 0.11 | -0.45 (0.44) | 1.11 (0.54) | **1.56 (0.69)** | **0.036** |
| Delivery System Design | 0.01 (0.37) | 1.06 (0.45) | 1.05 (0.58) | 0.07 | -0.08 (0.36) | 1.36 (0.43) | **1.44 (0.56)** | **0.011** |
| Clinical Information | 0.12 (0.42) | 1.29 (0.51) | 1.18 (0.66) | 0.08 | -0.07 (0.41) | 1.33 (0.50) | **1.40 (0.65)** | **0.033** |
| **Relational**  **Coordination**  **Overall** | 0.37 (0.17) | 0.10 (0.21) | -0.27 (0.27) | 0.31 | 0.35 (0.18) | 0.08 (0.21) | -0.26 (0.28) | 0.34 |
| Frequent | 0.32 (0.20) | 0.36 (0.24) | 0.04 (0.31) | 0.90 | 0.26 (0.21) | 0.21 (0.25) | -0.05 (0.33) | 0.89 |
| Timely | 0.30 (0.20) | -0.15 (0.24) | -0.45 (0.32) | 0.16 | 0.30 (0.20) | -0.02 (0.25) | -0.32 (0.32) | 0.32 |
| Accurate | 0.38 (0.20) | -0.15 (0.25) | -0.53 (0.32) | 0.10 | 0.37 (0.20) | -.11 (0.25) | -0.48 (0.32) | 0.14 |
| Problem Solving | 0.35 (0.22) | -0.02 (0.26) | -0.37 (0.34) | 0.27 | 0.30 (0.22) | -0.10 (0.27) | -0.40 (0.35) | 0.25 |
| Goals | 0.14 (0.21) | 0.07 (0.25) | -0.08 (0.33) | 0.82 | 0.11 (0.22) | 0.04 (0.26) | -0.07 (0.34) | 0.85 |
| Knowledge | 0.28 (0.20) | 0.04 (0.24) | -0.24 (0.31) | 0.45 | 0.25 (0.21) | -0.02 (0.25) | -0.27 (0.32) | 0.40 |
| a. Unadjusted estimates, clustered by site  b. Least Squares Means differences between time points within arms  c. Difference in difference analysis comparing post to pre intervention between arms (ICAN vs. Standard Care)  d. Adjusted for fixed effects of Healthcare professional age, sex, type of health professional, years in position/practice, Percentage of patient panel has chronic conditions, and random effect of site. | | | | | | | | |
