# Supplementary material for: Care for patients living with chronic conditions using the ICAN Discussion Aid: A mixed methods cluster-randomized trial
Source: PLoS One. 2024 Dec 4;19(12):e0314605. doi: 10.1371/journal.pone.0314605 (PMC11616879; doi:10.1371/journal.pone.0314605)
Supplement: S1 Appendix — (PDF) [file pone.0314605.s004.pdf]

Health Professional Surveys

- *Assessment of Chronic Illness Care (ACIC):*
  - Higher scores are better, range 0-11
  - "The ACIC provides subscale scores corresponding to each of the Chronic Care Model elements, as well as an overall score. Scores for each section are obtained by summing the values for all items within a section (e.g., self-management support) and dividing by the number of items within that section. The overall score is derived by summing the average scores of each section and dividing by the number of sections administered. For Version 3, you should divide the overall score (sum of average subscale scores) by 6 (the number of subscales in Version 3) to obtain the average overall score."[1]
  - The original paper with subscale scores demonstrated a range of mean (SD) scores from 3.16 (1.88) to 7.0 (1.27).[2]
  - Sensitivity to change has been demonstrated within scales ranging from mean change (SD) 1.89 (2.07) – 1.34 (2.29)[3]
- *Relational Coordination:*
  - Higher scores are better
  - Scale and scoring guide is proprietary and cannot be shared[4]

Patient in-Person Surveys

- *Self-efficacy to Communicate with Clinician*
  - Higher scores are better, range 1-10
  - 3 items, average score of 7.3, SD 2.71[5]
  - "The score for each item is the number circled. If two consecutive numbers are circled, code the lower number (less self-efficacy). If the numbers are not consecutive, do not score the item. The score for each scale is the mean of the items. For scales of 1-2 items, do not score the scale if any item is missing; for scales with 3-4 items, do not score the scale if more than 1 item is missing; for scales with 5-6 items, do not score the scale if more than 2 items are missing. Higher number indicates higher self-efficacy."[5]
- *Self-efficacy to Manage Chronic Disease*
  - Higher scores are better
  - Scale contains 6 items and the scores range from 1-10. Scoring is the following: "The score for each item is the number circled. If two consecutive numbers are circled, code the lower number (less self-efficacy). If the numbers are not consecutive, do not score the item. The score for the scale is the mean of the six items. If more than two items are missing, do not score the scale. Higher number indicates higher self-efficacy."[5]
  - The original study demonstrated a mean of 5.17 with standard deviation of 2.22.[5]
  - Mean change in follow up studies 0.36 – 0.84, with effect sizes of 0.16 – 0.38.[6]
- *Treatment Burden*

- Lower scores are better, range 0-150
- "It is composed of 15 items rated on a Likert scale ranging from 0 (not a problem) to 10 (big problem) related to the burden associated with taking medicine, self-monitoring, laboratory tests, physician visits, need for organization, administrative tasks, following advice on diet and physical activity, and social impact of the treatment. The total score is 150."[7]
- In the only study in which TBQ was used as an outcome, the statistically significant change in TBQ was reported as a mean difference of -5.0 with standard error of 2.[8]

- *Patient-Clinician Partnership measured using the Consultation Care Measure*
  - Lower scores are better, range of 21-147
  - "Participants completed a short questionnaire before their consultation in which they were asked to agree or disagree on a seven point Likert scale (very strongly agree to very strongly disagree) with statements about what they wanted the doctor to do."[9]
  - Subscales are a sum of the items; the overall score is a sum of all items. [9]

Patient Postal Surveys

- *Patient Assessment of Chronic Illness Care*
  - Each scale is scored by averaging the items completed within that scale, and the overall PACIC is scored by averaging scores across all 20 items. These scales emphasize patient-healthcare team interactions and, in particular, aspects of self-management support (e.g., goal setting, problem-solving)[10]
  - The mean (SD) PACIC in the original development paper was 2.60 (1.0)[11]
  - Mean change of 0.2 in another intervention study[12]
- *General Health assessed with a single item from the SF-36*
  - Lower score is better, range 1-5
  - In a systematic review of the measure, "In the pooled analysis, compared with persons reporting ''excellent'' health status, the odds ratio [95% CI] of mortality was 1.23 [1.09,1.39], 1.44 [1.21,1.72], and 1.92 [1.64,2.25] for individuals reporting ''good,'' ''fair,'' and ''poor'' health, respectively.[13]
- *Disruption from Illness and Treatment, assessed with the Illness Intrusiveness Scale*
  - Lower scores are better, range 13-91
  - The IIRS comprises 13 items that ask respondents to rate the degree to which their "illness and/or its treatment" interfere with life domains central to quality of life. Respondents employ a seven-point scale, ranging from 1 (not very much) to 7 (very much), to rate illness intrusiveness. When respondents consider an item not to be applicable (e.g., people who are no longer sexually active often identify the item tapping illness intrusiveness into "sex life" as not applicable), a score of "1" (one) is entered, indicating that the illness and/or its treatment do not interfere very much with this life domain."[14]
  - Means (SD) across a variety of conditions range from 16.2-55.3[14]

- Mean (SD) changes of -0.244 (0.841) have been reported as significant in other studies[15]

References

1. Accelerating care transformation (ACT) Center. Resource Library 2024 [cited 2024 June 19]. Available from: https://www.act-center.org/resources#2.
2. Bonomi AE, Wagner EH, Glasgow RE, VonKorff M. Assessment of Chronic Illness Care (ACIC): A Practical Tool to Measure Quality Improvement. Health Services Research. 2002;37(3):791-820. doi: https://doi.org/10.1111/1475-6773.00049.
3. Cramm JM, Strating MM, Tsiachristas A, Nieboer AP. Development and validation of a short version of the Assessment of Chronic Illness Care (ACIC) in Dutch disease management programs. Health Qual Life Outcomes. 2011;9:49. Epub 20110704. doi: 10.1186/1477-7525-9-49. PubMed PMID: 21726439; PubMed Central PMCID: PMCPMC3141373.
4. The Heller School for Social Policy and Management Brandeis University. What is Relational Coordination? 2024 [cited 2024 June 19]. Available from: https://heller.brandeis.edu/relational-coordination/about-rc/index.html.
5. Self-Management Resource Center. English Evaluation Tools 2024 [cited 2024 June 19]. Available from: https://selfmanagementresource.com/resources/evaluation-tools/english-evaluation-tools/.
6. Ritter PL, Lorig K. The English and Spanish Self-Efficacy to Manage Chronic Disease Scale measures were validated using multiple studies. J Clin Epidemiol. 2014;67(11):1265-73. doi: https://doi.org/10.1016/j.jclinepi.2014.06.009.
7. Tran VT, Montori VM, Ravaud P. Is My Patient Overwhelmed?: Determining Thresholds for Acceptable Burden of Treatment Using Data From the ComPaRe e-Cohort. Mayo Clin Proc. 2020;95(3):504-12. Epub 20191013. doi: 10.1016/j.mayocp.2019.09.004. PubMed PMID: 31619365.
8. Tinetti ME, Naik AD, Dindo L, Costello DM, Esterson J, Geda M, et al. Association of Patient Priorities-Aligned Decision-Making With Patient Outcomes and Ambulatory Health Care Burden Among Older Adults With Multiple Chronic Conditions: A Nonrandomized Clinical Trial. JAMA Intern Med. 2019;179(12):1688-97. doi: 10.1001/jamainternmed.2019.4235. PubMed PMID: 31589281; PubMed Central PMCID: PMCPMC6784811.
9. Little P, Everitt H, Williamson I, Warner G, Moore M, Gould C, et al. Observational study of effect of patient centredness and positive approach on outcomes of general practice consultations. Bmj. 2001;323(7318):908-11. doi: 10.1136/bmj.323.7318.908. PubMed PMID: 11668137; PubMed Central PMCID: PMCPMC58543.
10. University of Nebraska Medical Center. Patient Assessment of Care for Chronic Conditions 2024 [cited 2024 June 19]. Available from: https://www.unmc.edu/centric/_documents/R_V5.pdf.
11. Glasgow RE, Wagner EH, Schaefer J, Mahoney LD, Reid RJ, Greene SM. Development and validation of the Patient Assessment of Chronic Illness Care (PACIC). Med Care. 2005;43(5):436-44. doi: 10.1097/01.mlr.0000160375.47920.8c. PubMed PMID: 15838407.
12. Frei A, Senn O, Chmiel C, Reissner J, Held U, Rosemann T. Implementation of the chronic care model in small medical practices improves cardiovascular risk but not glycemic control. Diabetes Care. 2014;37(4):1039-47. Epub 20140210. doi: 10.2337/dc13-1429. PubMed PMID: 24513589.
13. DeSalvo KB, Bloser N, Reynolds K, He J, Muntner P. Mortality prediction with a single general self-rated health question. A meta-analysis. J Gen Intern Med. 2006;21(3):267-75. Epub 20051207. doi: 10.1111/j.1525-1497.2005.00291.x. PubMed PMID: 16336622; PubMed Central PMCID: PMCPMC1828094.
14. Devins GM. Using the illness intrusiveness ratings scale to understand health-related quality of life in chronic disease. J Psychosom Res. 2010;68(6):591-602. Epub 20090717. doi: 10.1016/j.jpsychores.2009.05.006. PubMed PMID: 20488277.
15. Lorig KR, Ritter PL, Dost A, Plant K, Laurent DD, McNeil I. The Expert Patients Programme online, a 1-year study of an Internet-based self-management programme for people with long-term conditions. Chronic Illn. 2008;4(4):247-56. doi: 10.1177/1742395308098886. PubMed PMID: 19091933.
