# Supplementary material for: Care for patients living with chronic conditions using the ICAN Discussion Aid: A mixed methods cluster-randomized trial
Source: PLoS One. 2024 Dec 4;19(12):e0314605. doi: 10.1371/journal.pone.0314605 (PMC11616879; doi:10.1371/journal.pone.0314605)
Supplement: S2 Appendix — (PDF) [file pone.0314605.s005.pdf]

An interactive version of the 16 survey items, from which this focus group guide was created, can be seen here: http://www.normalizationprocess.org/npt-toolkit/.

1. In what ways do you see ICAN as different from the way you normally work? In what ways do you see it as the same?
2. What would you say is the purpose of ICAN?
3. What do you understand ICAN requires of you?
4. What value do you see in ICAN for your practice?
5. Who are the key individuals for driving ICAN forward in practice? What sort of buy-in do you have from those individuals?
6. How strongly do you feel that ICAN should be part of your work?
7. How much do you buy-in to the potential impact of ICAN on the practice?
8. How supportive are you of ICAN?
9. What tasks would you say are required to implement ICAN in practice? Do you feel comfortable performing those tasks?
10. What different expertise do we have in the room? How might that expertise be applied differently to ICAN? Do you trust your colleagues to share the ICAN information amongst the care team?
11. Do you feel the work of ICAN is adequately distributed across the team?
12. What sort of buy-in do you feel from your organization to support ICAN?
13. How will you /have you determined if ICAN is effective in your practice? What have you learned from that information?
14. Do you feel that ICAN is overall a worthwhile endeavor as a team? What makes you think that?
15. Do you feel that ICAN is overall a worthwhile endeavor for your individual practice? What makes you think that?
16. What feedback loops have you received from using ICAN? What have you done with that information?

What barriers might there be to ICAN?

For each questions, use probes to get multiple perspectives in the room such as:

Are there other opinions?

Tell me what you think [name]?
