# Supplementary material for: Care for patients living with chronic conditions using the ICAN Discussion Aid: A mixed methods cluster-randomized trial
Source: PLoS One. 2024 Dec 4;19(12):e0314605. doi: 10.1371/journal.pone.0314605 (PMC11616879; doi:10.1371/journal.pone.0314605)
Supplement: S3 Appendix — (PDF) [file pone.0314605.s006.pdf]

ICAN Patient Interview Guide 2.0

We are interested in learning about how your overall visit went today including how you and your clinician used the ICAN tool in front of you. If you or your clinician didn't use ICAN today, that is ok, we can still learn a lot from you.

1) Let's start with your visit today, can you give me an overview of what you and your clinician discussed today?

   Probes:

   - What was the original reason you made the appointment?
   - Were those (original reasons) the main things discussed during the appointment?

   If you didn't get much info:

   - What did you discuss at the beginning of your visit?
   - What did you discuss in the middle of the visit?
   - And how did you all end the visit?

2) In what ways were you pleased with the way your conversation went?

3) In what ways were you frustrated with the way your conversation went?

4)
5) How does this visit compare to previous visits with this clinician? [OMIT IF PATIENT IS NEW PATIENT TO CLINICIAN]

   Probes:

   - Different or similar conditions addressed?
   - Different or similar in terms of the reason for the appointment?
   - Different or similar topics of discussion?

6) How does this visit compare to visits with other clinicians?

   Probes:

   - Similar or different in topics of discussion?
   - Similar or different in the clinician's style?

7) What, if any, services in your community or other clinicians has Dr. XX referred you to in the past?

   Probes:

- Has he/she referred you to community services such as transportation, home healthcare services, community health programs, etc.?
- If so, what were those services?
- What was the reason for that/those referral(s)?
- Has he/she referred you to other clinicians, such as specialists for various conditions you have?
- If so, what types of clinicians were you referred to?
- What was the reason for that/those referral(s)?

8) Now, I want to talk to you a little bit about the ICAN tool. Can you tell me what you thought about as you were filling it out?

   If patient filled, probes:

   - What did you think about when you were filling in this side? → point to left (life) column
   - What did you think about when you were filling out this side? →point to right (tx) column
   - What, if any, ideas or thoughts did using this tool bring to mind that you wouldn't have normally thought about before your appointment?
   - What, if any, ways did this make you feel more prepared for your appointment?
   - Did using this card change anything about your visit from a typical visit? If so, what was that? If not, in what situations do you think it might be helpful?

   If patient did NOT fill, probes:

   - Would you mind filling it out with me and telling me what you're thinking while we go?
   - Now that you have filled it out, can you tell me how you think you might use this with your clinician in the future? What else would you like to share about how you used the tool before your visit?

9) Ok, now I would like to ask how your clinician used the tool. Did your clinician look at the card during your visit?

➔ If yes, proceed with the remainder of questions.
➔ If no, proceed to question 13.

10) Ok great, would you mind walking me through what they did with the tool?

    Probes:

    - Did they ask you what stood out to you from filling the tool?
    - Did they ask you about specific things you listed?
    - How did they connect that to the care you received today?
    - What, if any, suggestions or recommendations did your clinician make as a result of using the tool?

11) In what way were you pleased by the way your clinician used the tool today?

12) In what way were you frustrated by the way your clinician used the tool today?

13) How does your clinician usually start your visit? Is it similar or different from how your visit began today?

14) I am going to list three questions for you that your clinician may have used during your visit. Can you tell me if you remember him/her using any of these?

    1) What are you doing when you're not sitting here with me?
    2) Where do you find the most joy in your life?
    3) What is on your mind today?

15) Do you have any ideas you would like us to consider incorporating in the ICAN tool to make it better for patients like yourself?

16) Is there anything you would like us to change, add, or delete from this tool to make it better?

I would like to thank you for your time! This has been very helpful.
