# Supplementary material for: Care for patients living with chronic conditions using the ICAN Discussion Aid: A mixed methods cluster-randomized trial
Source: PLoS One. 2024 Dec 4;19(12):e0314605. doi: 10.1371/journal.pone.0314605 (PMC11616879; doi:10.1371/journal.pone.0314605)
Supplement: S4 Appendix — (PDF) [file pone.0314605.s007.pdf]

Explicit Statements
Elements of ICAN/Keywords/Phrases (especially when used together)

Explicit Statements:

- My Life, My Healthcare
- ICAN
- Mayo Clinic
- Communication Tool
- Discussion Aid

Elements of ICAN:

- Areas of Life:
  - Family/Friends
  - Work
  - House/Neighborhood
  - Finances
  - Free Time/Relaxation/Fun
  - Faith/Personal Meaning
  - Being Active
  - Rest/Comfort
  - Emotional Life
  - Senses/Memory
  - Eating Well
- Clinician Questions:
  - What are you doing when you're not sitting here with me?
  - Where do you find the most joy in your life?
  - What's on your mind today?

Keywords/Phrases:

- Capacity
- Burden
- Satisfaction
- "Difficulties with…"
- "Barriers to…"
- Work (in the context of the work of being a patient)
- "Things we've asked the patient to do…"
- "Sources of…[joy/capacity/etc.]"
- Cope/coping

Examples of text when ICAN was used, as document in the EMR:

In regard to her diabetes, she sometimes "gets tired" of being a diabetic but she understands the importance of keeping her diabetes under good control. She is okay to continue with five injections daily and is not interested in pursuing use of an insulin pump at this time stating that she is hypersensitive to insulin and sugars. Her plans to help improve her diabetes include increasing her physical activity which is always easier for her to do a nice weather. Her most recent hemoglobin A1c is 8.5. She is previously followed with an endocrinologist downtown however her endocrinologist has retired and she is now seeing an NP for her endocrine care she would ideally like to simplify care and is much as possible at this office location. She has a good social support system and has written the following comments for me:
"Chronic illness is just a different type of lifestyle the normal which one has to learn to accept and adjust to accordingly to feel good each day after being quite ill one does not want to return there".

Feels her sister, who obtained the phentermine, through "not the right ways," per her report. Is not what she would like to do. She would like to do it under my supervision and guidance. Feels that use of a pill is her best option at this time. Admits to several stressors, particularly in the last month, for her and her family. Her father suffered a stroke. Her niece was admitted to [redacted]. She has been caring for her two nieces and her niece's son while her sister and brother-in-law are out of the country. Feels sleep is not ideal. Says "it will get better eventually." When asked what she does that is enjoyable to her, she cannot find anything that she does that is enjoyable and feels all this will become better when her children get older. Denies feeling depressed but was tearful during discussion of her weight, what she enjoys in life, and discussion of how to make lifestyle changes and also specifically smoking cessation. Denies any thoughts of harming herself or anyone else.

Ms. [redacted] admits that she has not been focusing her efforts on her diabetes. However, she has joined the Y gym with her sister and tries to exercise on a regular basis. She has lost 2 kg, and she is very happy with that. She admits that checking her blood glucose and her insulin therapy is a burden for her at this point.
